# Effects of Different Scratch Mat Designs on Hen Behaviour and Eggs Laid in Enriched Cages

**DOI:** 10.3390/ani11061544

**Published:** 2021-05-25

**Authors:** Victoria Sandilands, Laurence Baker, Jo Donbavand, Sarah Brocklehurst

**Affiliations:** 1 Easter Bush Campus, Scotland’s Rural College (SRUC), Midlothian EH25 9RG, UK; Laurence.Baker@sruc.ac.uk (L.B.); Jo.Donbavand@sruc.ac.uk (J.D.); 2 BioSS, JCMB, The Kings Buildings, Peter Guthrie Tait Road, Edinburgh EH9 3FD, UK; Sarah.Brocklehurst@bioss.ac.uk

**Keywords:** laying hen, foraging, dustbathing, furnished cages, egg quality

## Abstract

**Simple Summary:**

Enriched cages for laying hens must contain litter so that pecking and scratching are possible. This is typically provided using layer’s feed dispensed onto a scratch mat, however, there are no regulations on the size or materials of the mat. This study examined how different scratch mat designs and bird age affected behaviours on the mat at three times of day, and their influence on where eggs were laid and shell quality. The proportion of hens at the scratch mats did not increase during or shortly after the application of scratch feed, however, they were more likely to be foraging then. Most eggs collected were clean and laid in the nest. Of the small proportion of eggs that were cracked or dirty, the mat type did not affect dirty eggs, but eggs laid opposite the Big Dutchman mats were more likely to be cracked at 79 weeks of age than at any other mat type or age. There appeared to be no optimal scratch mat design (of those studied) and their use (during observations) was low, suggesting that mat designs were not major influencers on bird behaviour.

**Abstract:**

Laying hens in the UK and EU must be provided with litter for pecking and scratching. In enriched cages, this is commonly provided by dispensing layer’s feed onto a scratch mat. Mats vary in design and size, which might affect hen behaviour and egg quality, since eggs are sometimes laid at the mats. We investigated if four different scratch mats (BD, K, V, Z) provided to hens in enriched cages resulted in differences in behaviour on the mats and external egg quality. Twenty-four 60-bird cages (6 cages/bank × 4 banks) with 2 mats/cage at one tier of a commercial enriched cage unit were used. Mats were allocated to cages in a balanced design prior to the flock arriving. Hens and eggs were studied at 30, 50 and 79 weeks of age, with three behaviour observations (before, during or after scratch feed application). The data were analysed by GLMMs or LMMs. The vast proportions of birds on the mats were standing (0.720) or sitting (0.250). Bird proportions on the mats were low overall and declined from 0.028 (30 weeks) and 0.030 (50 weeks) to 0.020 (79 weeks) (*p* < 0.001). The greatest proportion of hens were observed on Z (*p* < 0.001), which had the largest area, but relative to the available area least birds were on Z and most were on K (*p* < 0.001). Foraging was not affected by bird age or mat type but was greater at the second observation (*p* < 0.001). Most eggs were laid in the nest box and were clean. Clean eggs declined, and dirty eggs increased, significantly with age, particularly at the scratch mat (*p* < 0.001). Dirty eggs were not affected by mat design. Cracked eggs were highest at 79 weeks of age, particularly with BD mats (*p* < 0.001). Overall, scratch mat designs had minimal effects on behaviour (but few hens were seen there) and egg quality.

## 1. Introduction

Enriched cages (sometimes referred to as furnished cages) are the only permitted method of housing laying hens in cages in the EU and UK [1]. Although they are falling out of favour in some countries, in others they are still in common use: in 2020, enriched cage eggs made up 40% of eggs through UK packing stations [2]. The benefits of enriched cages over conventional (battery) cages are that they provide more space per hen and enrichments such as a nesting area, perches, and litter so that pecking and scratching are possible.

There are two main methods used to provide litter. One is to put litter into a dustbath, used in research cage prototypes [3] and some commercial cage designs (e.g., Specht, [4,5]; Victorrson, [6]; Big Dutchman, [7]). However, this method can result in eggs being laid there [8] and the litter (woodshavings, sand) can interfere with the auger mechanisms. The other design is to put a mat on top of the wire floor, onto which layer’s feed is dispensed (e.g., Zucami, [9]; Big Dutchman, [10]). Eggs may still be laid there, but they can roll freely onto the egg belt. In either method of litter provision, hens may use the litter to express dustbathing or foraging behaviours [9,11,12].

Foraging is an important natural behaviour in laying hens. It includes food-seeking behaviours such as ground pecking and scratching [13]. In a study of captive-bred jungle fowl (the ancestors of domesticated chickens), hens were seen ground pecking for 60.6% and ground scratching for 34.1% of observations, respectively (although behaviours were not mutually exclusive) [14]. In a group of feral bantam chickens observed over 8 months, the proportion of time observed feeding was 47.9%, with an average of 50.4 pecks/min [15]. Laying hens that do not have opportunities to forage may develop feather pecking, a serious welfare concern that can lead to severe feather loss, cannibalism and even death [16]. Feather pecking is thought to be related (in part) to the inappropriate redirection of foraging behaviour onto the feathers of other hens, usually where suitable substrates for pecking and scratching are not available [17,18]. In commercial environments, laying hens have various opportunities to show pecking and scratching behaviour, depending on the resources available. In alternative systems such as those used in barn, free-range and organic egg production, where at least one third of the floor is litter [1] which is continuously present, there are arguably more opportunities to show pecking and scratching than in enriched cages, where the scratch mat is small, and litter is not always present due to its being dispersed by the birds. This is compounded because the legislation does not stipulate how to present the litter in cages. As a result, scratch mats in enriched cages come in a variety of designs and sizes, which may affect hens’ abilities to express foraging behaviour there. Furthermore, although nests are provided in enriched cages, scratch mats can be attractive for egg laying, particularly if competition for nest space is high [19]. Laying eggs on the scratch mats (or at least, outside of the nest box) can affect the proportion of dirty eggs produced, particularly if mats are dirty with excreta [20,21]. It may be that different designs of scratch mats will affect external egg quality in different ways, because they can be made of different materials that might be more likely to damage eggs or hold excreta. The aim of this study was to examine the effects of four different scratch mat designs on the behaviour of commercial hens housed in an enriched cage system. We investigated behaviours on the mats, where eggs were laid and the external shell quality.

## 2. Materials and Methods

### 2.1. Hens and Cages

**Figure 1:**
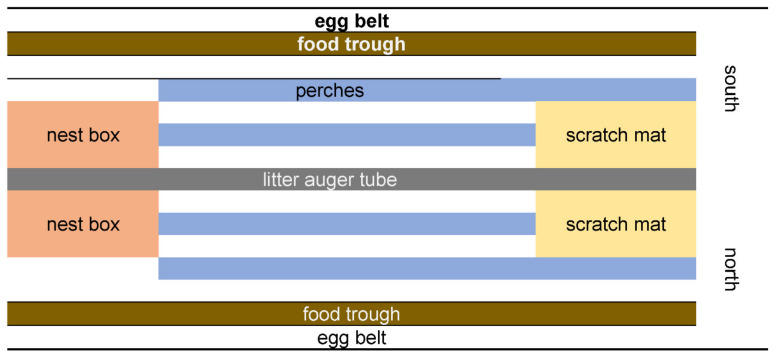
Overhead view of one enriched cage (not to scale), with furniture (nest boxes, perches, and scratch mats) locations noted. Cages spanned an entire bank width, and so cage sides were referred to as north or south.

A flock of Hy-Line Brown laying hen pullets was sourced from a single rearing farm and placed into enriched cages at 15–16 weeks of age. The laying hen farm was located in Scotland and consisted of 1674 60-bird enriched cages (Big Dutchman, Vechta, Germany), over six banks and nine tiers, with 31 cages/tier. Each cage spanned the entire bank width, and contained two nest boxes, perches (15 cm/hen) and two scratch mats, one on either side of the bank (north and south) (Figure 1).

**Figure 2:**
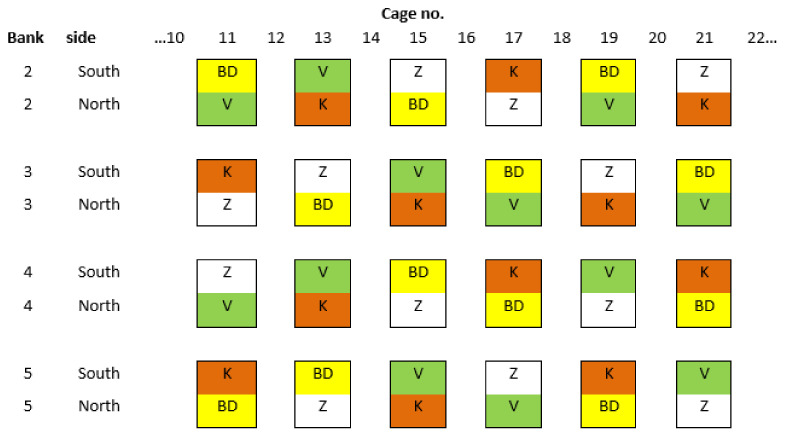
Overhead diagram of scratch mat treatment layout per cage in tier 5, at banks 2, 3, 4 and 5. BD = Big Dutchman, K = Kovobel, V = Valli, Z = Zucami design mats. There was one scratch mat on each side of the cage (north and south). Cage no. = cage number.

For this study, 24 cages were used in banks 2–5 at tier 5 (6 cages/bank) (Figure 2). Every other cage was used from cage 11–21, so that there was no cross-contamination of eggs with neighbouring cages for egg quality data. Prior to pullets arriving, some of the original mats were replaced with other mat types (K = Kovobel^®^, Domazlice, Czech Republic; V = Valli^®^, Italy; or Z = Zucami^®^ Poultry Equipment, Beriain, Spain) in a balanced design, so that each mat was equally represented across banks, cage locations and cage sides.

**Figure 3:**
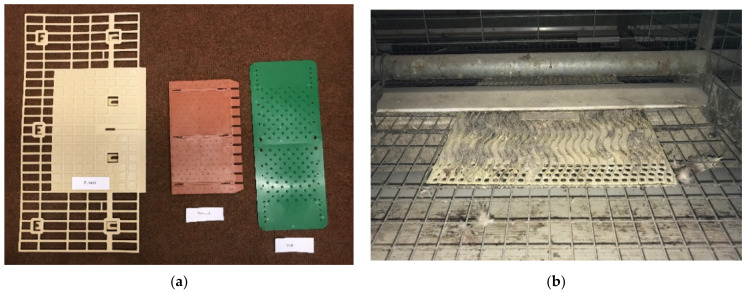
Image of the three test scratch mats (**a**) (from left to right) Zucami, Kovobel, and Valli and (**b**) the in situ mat (Big Dutchman); note the auger tube directly above, which provides the layer’s feed as ‘litter’.

Where the original mats (which had claw shorteners on them) were removed, claw shorteners were stuck to the feed baffle on that cage side, to stay within the regulations. Mats varied in size and colour but were all located in the original mat position so that feed from the litter auger tube would fall onto the mat when activated (Figure 3). Lights came on at 04:00, with first feed at 04:35 until 72 weeks of age, when an extra hour of light was added (lights on 03:00, first feed 03:35). Layer’s mash was applied as litter (scratch feed) through the litter auger tube at 04:35 (03:35 from 72 weeks), 09:20 and 14:20. Lights went off at 18:00. As a result of their various designs, the mats provided different amounts of scratch mat space per hen and different relative areas (Table 1).

### 2.2. Behaviour

Hens were observed at three ages (30, 50 and 79 weeks of age). At each age, hen behaviour at the mats was observed three times. The first (1st) observation was at 11:00 (30 weeks), 11:30 (50 weeks) or 08:20 (79 weeks) which equated to 1 h 40 min, 2 h 10 min, or 4 h 45 min since last scratch feed provision respectively, and was conducted before the observed scratch feed application; the second (2nd) observation was during and immediately after scratch feed application (14:20 for both 30 and 50 weeks, 09:20 for 79 weeks) but only observed half of the cages (balanced for mat types) to capture behaviour when scratch feed was most likely to be present; the third (3rd) observation was at 15:00 (30 and 50 weeks) or 10:20 (79 weeks) which equated to 40 min or 1 h after the observed scratch feed application. A total of 360 observations (24 cages × 2 sides/cage × 3 visits × 2.5 observations/visit) were carried out, using scan sampling methods to count the number of hens performing behaviours on the mats. The proportions of hens on the scratch mat engaged in behaviours were calculated (number of hens seen on scratch mat/total hens in the cage, or number of hens engaged in particular behaviour on the mat/total hens seen on the mat) (Table 2).

### 2.3. Eggs

In enriched cages, to preserve egg quality, eggs roll forward from the nest box and rest against an ‘egg saver’ wire under the feed trough. This lifts at regular intervals set by the farmer to allow the eggs to roll further forward onto the egg belt, which is stationary. At intervals set by the farmer, the egg belt then nudges forward by approximately 1 m towards the egg elevators at the end of the bank, to prevent the build-up of eggs opposite the nest box. On the day before egg and behaviour observations, farm staff cleared the egg belts of all remaining eggs laid by that afternoon (most eggs are laid in the first few hours after lights on). On the day of egg assessment, in order to keep treatment cage eggs distinct from neighbouring cage eggs, neighbouring cage eggs that were on the egg belts since the previous day’s last egg saver wire lift (17:00) were removed. Immediately after the first egg saver wire lift at 05:30, treatment cage eggs were collected (from both sides of the cage) and placed on trays labelled according to the cage furniture area from whence they came (i.e., opposite the nest box, scratch mat, or any other area), while neighbouring cages’ eggs were simply removed. This was repeated a further three times when the egg saver wires lifted at 06:15, 07:00, and 07:45.

Treatment cage eggs were counted by location laid and by egg quality factors likely to be affected by cage furniture (dirty (with faeces), cracked, clean). The proportions of eggs by location and by egg quality out of the total eggs collected per cage were calculated.

### 2.4. Statistics

All data were compiled in Excel. Genstat 18 was used for data processing and all statistical analyses. To analyse proportions, Generalised Linear Mixed models (GLMMs) were fitted to binomial counts with appropriate binomial totals, logit link function, binomially distributed errors and dispersion fixed at 1. To analyse counts, Generalised Linear Mixed models (GLMMs) were fit to the counts with log link function, Poisson distributed errors and dispersion fixed at 1. Where data was sparse and GLMMs with all effects included would not converge random and fixed effects, these models were simplified. Linear Mixed models (LMMs) with all effects included were used as approximations in addition to simplified GLMMs for binomial data. With LMMs, proportion data were first angular transformed to degrees scale (see (1) below) to normalise the distribution of residuals, i.e., for proportion *p*:

(180/π)sin^−1^(*√p*) (1)

In the results, statistical analyses for the following measurements are reported:

- counts of birds observed on the mat (all behaviours) on each cage side at each behaviour observation with and without offset log relative area of the scratch mats.
- counts of birds on the mat on each cage side at each behaviour observation exhibiting different behaviours (standing, sitting, foraging) out of number of birds observed on the same mat at the time of the behaviour observation (thus all reported estimates in the results are on the scale of the proportion (or proportion transformed) of each behaviour on the mat, out of total birds on the mat).
- counts of egg types (clean, dirty, cracked) out of eggs of all types collected from the belt opposite each location in the cage side (nest, scratch, other) on egg assessment days (thus all reported estimates in the results are on the scale of the proportion (or proportion transformed) of each egg type, out of total eggs at each location).

In the LMMs for proportions of birds on the mat or behaviours, fixed effects were bird age (30, 50, 79), observation (1st, 2nd, 3rd), and mat type (BD, K, V, Z) and all 2- and 3-way interactions; random effects were bank, cage, cage.age, cage.age.observation and cage side within cage. In the LMMs for proportions of egg types, fixed effects were bird age (30, 50, 79), location (nest, scratch, other) and mat type (BD, K, V, Z) and all 2 and 3 way interactions; random effects were bank, cage position, side, cage, cage.age, cage.age.location and cage side within cage. Corresponding GLMMs were similar to these LMMs but with both fixed and random effects simplified as required depending on how sparse the data counts were for each response variable in order to achieve model convergence whilst retaining the most important effects. In GLMMs dispersion was fixed at 1. P values are based on approximate *F* tests when available but otherwise are based on Wald tests; statistics are given in the results as Wald**_ndf_** and F**_ndf,ddf_**, where ndf is the numerator degrees of freedom (the number of effects to be estimated, which is the number of levels for a categorical factor less 1) and ddf is the denominator degrees of freedom. Model estimates ± standard errors (SE) obtained from the LMMs and GLMMs are reported as well as estimates back transformed onto the original scale (proportion) to aid interpretation.

The data relating to this study have been deposited in the repository Zenodo (https://zenodo.org/, Geneva, Switzerland, accessed on 23 March 2021), access number md5:2bbc31c13aaf30f8e56ae2c3b5691b01.

This study was ethically approved by SRUC’s Animal Experiments Committee, number POU AE 11-2019.

## 3. Results

### 3.1. Behaviour

A total of 805 hens were observed on the mats over 360 observations (thus on average 2.2 hens/mat/observation). Of the total hens seen on the mat, the greatest proportion was seen standing followed by sitting, with 0.03 of hens seen engaging in forage, preen, or walk (Table 3). Hens were not observed dustbathing or in other behaviour.

#### 3.1.1. Counts of Birds on the Mats

The mean number of birds on the mats engaged in any behaviour was significantly affected by bird age: the count of birds was significantly lower at 79 weeks of age (0.181) than at 30 (0.518) or 50 (0.571) weeks of age (mean SE 0.097, *p* < 0.001 by GLMM, Wald_2_ = 24.18, back transformed counts (proportions of 60 birds shown in parentheses): 30 weeks 1.68 (0.028), 50 weeks 1.77 (0.030), 79 weeks 1.20 (0.020)). There was no significant effect of observation (i.e., 1st 2nd or 3rd) (*p* = 0.587).

The mat type significantly affected the proportion of birds on the mats, with a greater proportion of hens seen on the Z mats (0.798) than any other type (BD 0.338, K 0.273, V 0.284, mean SE 0.106, *p* < 0.001 by GLMM, Wald_3_ = 42.78, back transformed counts (proportions) BD 1.40 (0.023), K 1.31 (0.022), V 1.33 (0.022), Z 2.22 (0.037)). In the model which adjusts for mat areas, however, although mat type is still significant, more birds were on K and less on Z relative to the available area (BD 1.879, K 2.284, V 1.964, Z 1.562, SE 0.106, *p* < 0.001 by GLMM, Wald_3_ = 31.89, back transformed counts (proportions) BD 1.64 (0.027), K 2.46 (0.041), V 1.78 (0.030), Z 1.19 (0.020)). There were no significant interactions (age × observation, age × mat type, or observation × mat type) on the number of hens seen on the mats.

#### 3.1.2. Standing

For the proportion of hens seen standing on the mats (number of hens seen standing/total birds on the mat), there was a significant age effect (*p* < 0.001), with the number of hens standing declining with age (30 weeks 2.578, 50 weeks 1.362, 79 weeks 0.212, mean SE 0.283, by GLMM, Wald_2_ = 63.37, back transformed proportions 30 weeks 0.929, 50 weeks 0.796, 79 weeks 0.553). There was a weak effect of observation (*p* = 0.021), with a greater proportion of hens seen standing at the second observation (1.913) than at the first (1.066) or third (1.173) (mean SE 0.283, by GLMM, Wald_2_ = 7.75, back transformed proportions 1st 0.744, 2nd 0.871, 3rd 0.764). There was no significant effect of mat type on the proportion of hens seen standing on the mat (*p* = 0.589), nor any significant interactions from the GLMM or LMM.

#### 3.1.3. Sitting

**Figure 4:**
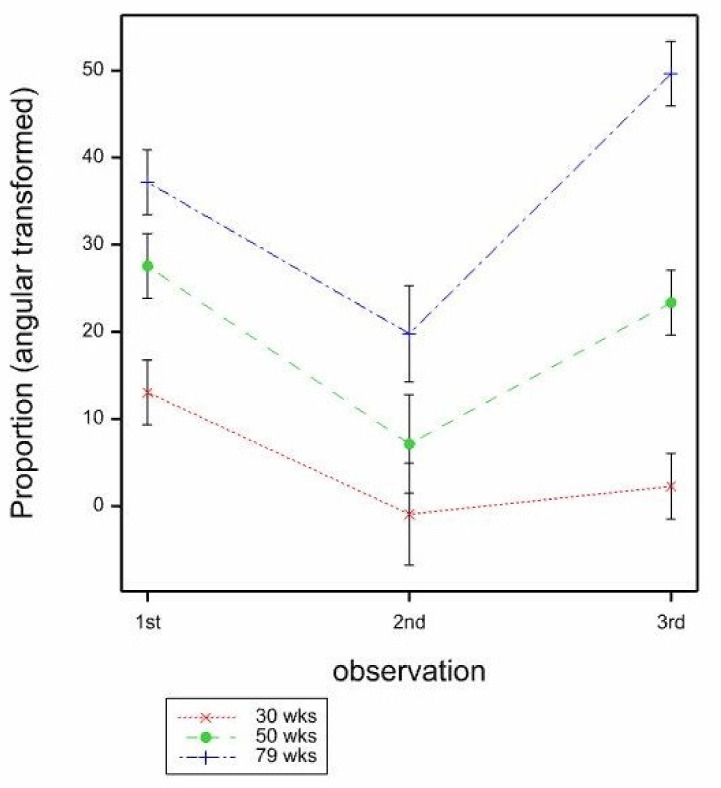
Mean proportions of hens sitting on the mats by age (30, 50, 79 weeks) and observation (1st, 2nd, 3rd) estimated from LMM. Mean SE 4.840.

For the proportion of hens seen sitting on the mats, there was a significant age effect (*p* < 0.001), with the proportion of hens seen sitting increasing with bird age (30 weeks −2.998, 50 weeks −1.741, 79 weeks −0.568, mean SE 0.266, by GLMM, Wald_2_ = 57.34, back transformed proportions 30 weeks 0.048, 50 weeks 0.149, 79 weeks 0.362). There was a significant effect of observation (*p* < 0.001), with a smaller proportion of hens observed sitting at the 2nd observation (−2.912) compared to the 1st (−1.169) and 3rd (−1.225) observations (mean SE 0.273, by GLMM, Wald_2_ = 20.85, back transformed proportions 1st 0.237, 2nd 0.052, 3rd 0.227). The mat type effect was not significant (*p* = 0.685) and there were no significant interactions for proportion of hens sitting, although the GLMM model failed for age × observation due to sparse data. The LMM analysis indicates that there was a significant, but weak, age × observation interaction (mean SE 4.840, *p* = 0.024, by LMM, F_4,154_ = 2.90) (Figure 4) with the proportion of hens sitting slightly greater for hens at 79 weeks of age at the third observation than at other ages, but fairly similar at other ages and observations. (In fact, generally when birds were observed on the mat, if they were not sitting, they were mostly standing, although the related age × observation for standing was not significant.)

#### 3.1.4. Foraging

**Figure 5:**
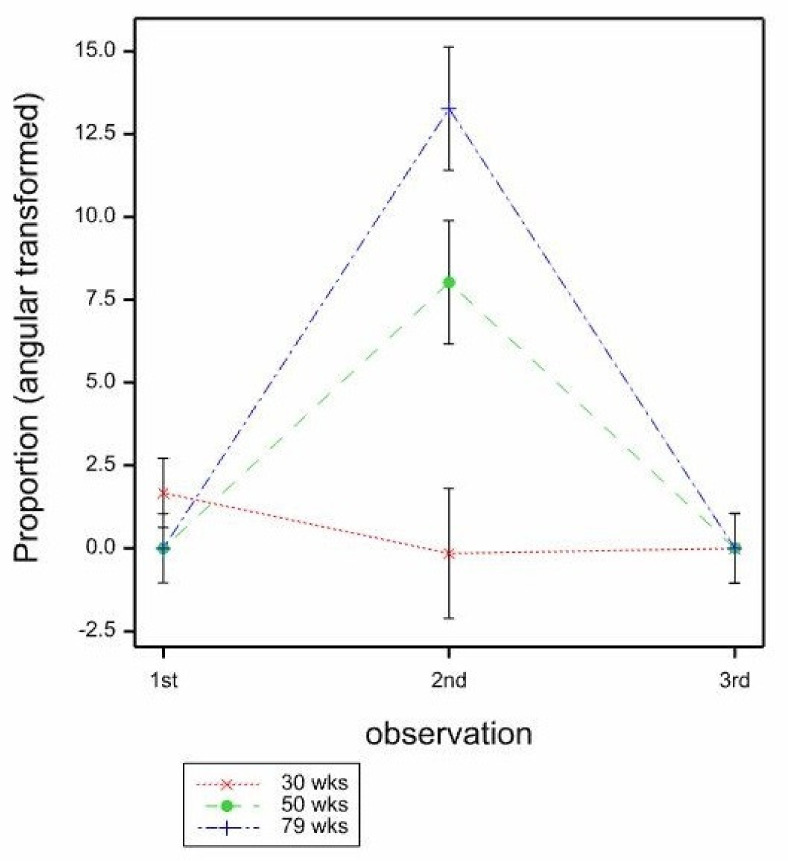
Mean proportions (angular transformed) of hens foraging on the mats by age (30, 50, 79 weeks) and observation (1st, 2nd, 3rd) estimated from LMM. Mean SE 1.808.

The proportions of birds foraging on the mats were very low (0.021 overall), however, this was an area of particular interest, to see if different mats stimulated more foraging than others. There were no significant effects of bird age (*p* = 0.512) or mat type (*p* = 0.892) on the proportion of birds foraging, by GLMM, however GLMM models with any other effects or interactions included failed to converge. When LMM was used, there was a significant effect of observation on the proportion of foraging seen (*p* < 0.001), with most at the 2nd observation (1st 0.557, 2nd 7.046, 3rd −0.001, mean SE 1.122, by LMM, F_2,133_ = 11.49, back transformed proportions 1st 0.000, 2nd 0.015, 3rd 0.000). The LMM indicated a significant age × observation interaction also (*p* = 0.002, mean SE 1.808, by LMM, F_2,146_ = 4.51), where the proportion of birds foraging was similarly low across ages at both the 1st and 3rd observation, but at the 2nd observation, more birds were seen foraging at 79, then 50, then 30 weeks (Figure 5).

Statistical results for other behaviours are not reported, due to their rare occurrence.

### 3.2. Eggs

A total of 3564 eggs were assessed from all studied cages over 3 ages. Of those, almost 89% were laid in the nest box, and only 3.9% were laid at the scratch mats, with just over 7% laid in other areas of the cage. The majority of eggs (96.5%) were clean, 1.9% were cracked and 1.6% were dirty (overall eggs from all cages studied).

**Figure 6:**
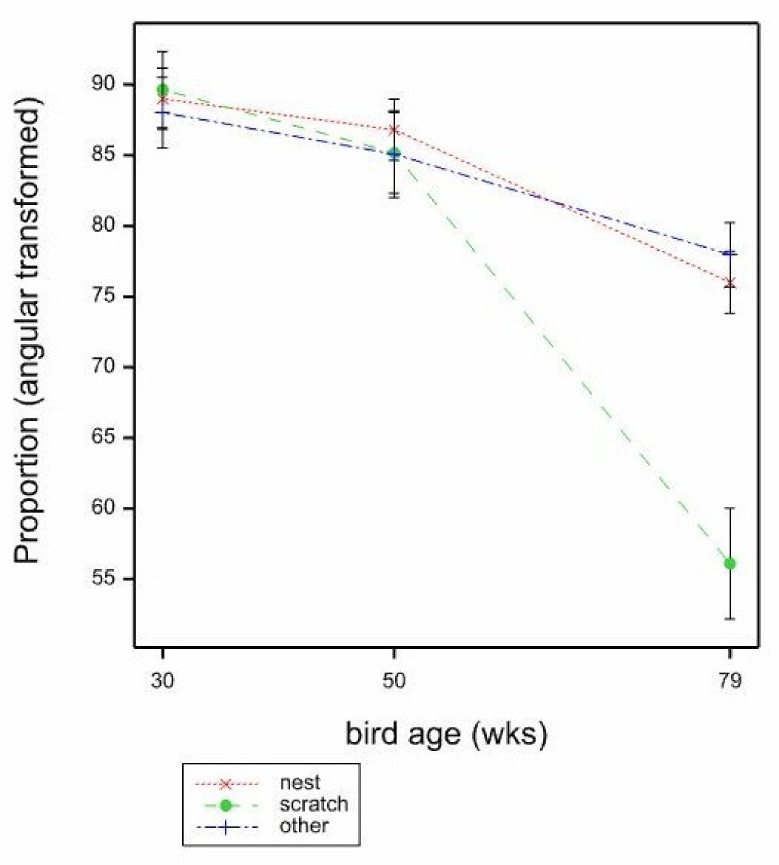
Mean proportions (angular transformed) of clean eggs per location according to bird age (30, 50, 79 weeks) and cage location (nest, scratch mat, other areas of the cage) estimated from LMM. Mean SE 3.566.

The proportion of clean eggs was significantly affected by bird age (Table 4), with the proportion of clean eggs per location declining with age (*p* < 0.001 by GLMM, Wald_2_ = 71.13, 30 weeks 4.871, 50 weeks 3.538, 79 weeks 1.791, mean SE 0.386, back transformed proportions 30 weeks 0.992, 50 weeks 0.972, 79 weeks 0.857). When comparing egg types per location between cage locations, a significantly greater proportion of clean eggs came from the nest, followed by other areas of the cage, and least proportion of clean eggs came from the scratch mat (*p* < 0.001, by GLMM, Wald_2_ = 28.89 Nest 4.223, Scratch 2.677, Other 3.300, Mean SE 0.377, back transformed proportions Nest 0.986, Scratch 0.936, Other 0.964) (Table 5). There was no effect of mat type (*p* = 0.699) or any interactions, although the GLMM model failed for bird age × location due to sparse data. However, bird age × location was significant (*p* < 0.001 by LMM) where the proportion of clean eggs per location was seen to be significantly lower at the scratch mat at 79 weeks than all other ages by location (F_4,252_ = 5.05, mean SE 3.566, Figure 6).

**Figure 7:**
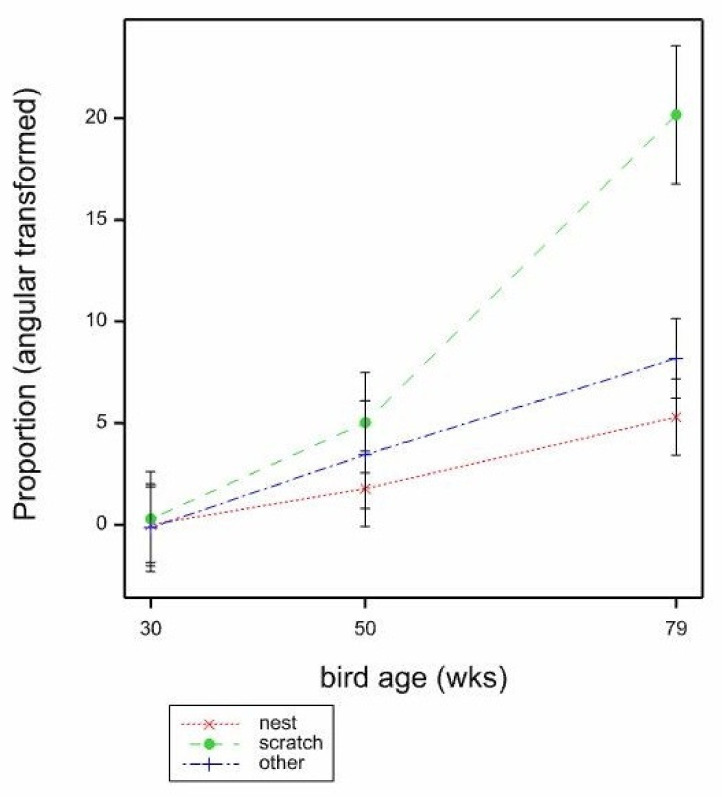
Mean proportions (angular transformed) of dirty eggs per location by bird age by location in the cage (nest, scratch mat, all other areas) estimated from LMM. Mean SE 3.219.

Statistics on the proportion of dirty eggs and cracked eggs should be treated with caution, as these are based on less than 2% each of all eggs assessed. For the proportion of dirty eggs per location, most GLMM analysis failed for this reason, however, from the GLMM including fixed effects of mat type and location only, there was a significant effect of location (*p* < 0.001), with the greatest proportion of dirty eggs per location laid at the scratch mat, and least at the nest (Nest-4.843, Scratch-2.711, Other-3.187, Mean SE 0.330, by GLMM, Wald_2_ = 40.83, back transformed Nest 0.008, Scratch 0.062, Other 0.040) (Table 5). There was no effect of mat type (*p* = 0.827). From the LMM, there was a significant effect of age (*p* < 0.001), with the proportion of dirty eggs per location increasing with age (30 weeks 0.047, 50 weeks 3.415, 79 weeks 11.218, Mean SE 2.586, F_2,59_ = 12.93, back transformed 30 weeks 0.000, 50 weeks 0.004, 79 weeks 0.038). There was also a significant effect of bird age × location on dirty eggs (*p* = 0.001, by LMM, mean SE 3.219, F_4,101_ = 5.0, Figure 7) with the proportion of dirty eggs per location similar at all cage locations at 30 and 50 weeks of age, but with significantly greater dirty eggs laid in the scratch at 79 weeks compared to the nest and other cage locations. All other interactions were not significant in the LMM.

**Figure 8:**
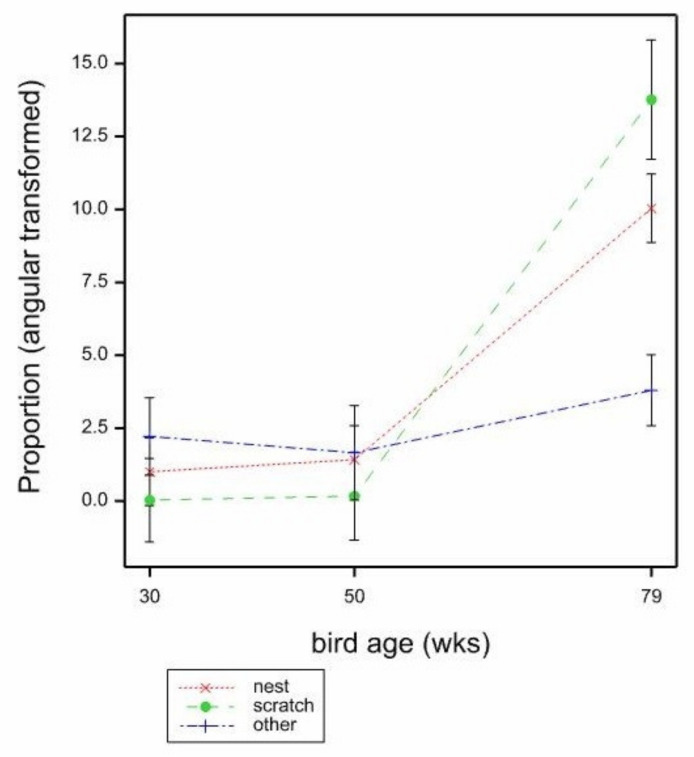
Mean proportions (angular transformed) of cracked eggs per location by bird age (weeks) and location in the cage (nest, scratch mat, all other areas) estimated from LMM. Mean SE 1.465.

**Figure 9:**
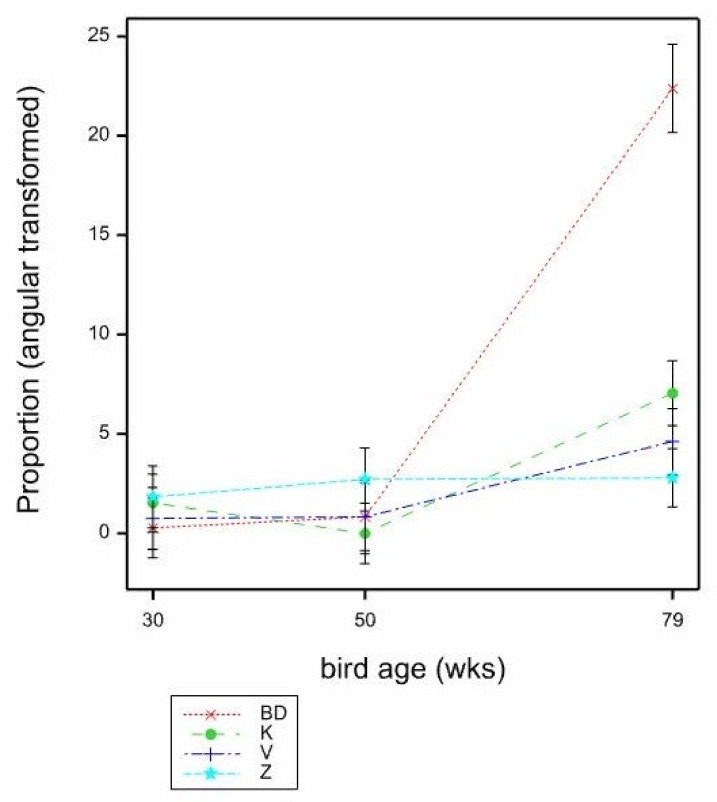
Mean proportions (angular transformed) of cracked eggs per location by bird age (weeks) and mat type estimated from LMM. Mean SE 1.691.

With the proportion of cracked eggs per location, there was a significant effect of age (*p* < 0.001 by GLMM, 30 weeks −5.256, 50 weeks −4.905, 79 weeks −2.942, Mean SE 0.3983, Wald_2_ = 49.07, back transformed 30 weeks 0.005, 50 weeks 0.007, 79 weeks 0.050) with a higher proportion of cracked eggs per location seen at 79 weeks. There was no significant effect of location or mat type. With interactions, all GLMMs failed, but LMMs indicated some significant interactions. There was a significant bird age × location effect (*p* = 0.016, by LMM, F_4,267_ = 3.11, mean SE 1.465) with a greater proportion of cracked eggs per location seen at the nest and scratch mat than at other areas of the cage at 79 weeks (Figure 8). There was a significant age × mat type effect (*p* = 0.002, by LMM, F_6264_ = 3.49, mean SE 1.691) with the proportion of cracked eggs per location significantly higher at 79 weeks opposite Big Dutchman (BD) mats, as opposed to any other mat type (Figure 9).

**Figure 10:**
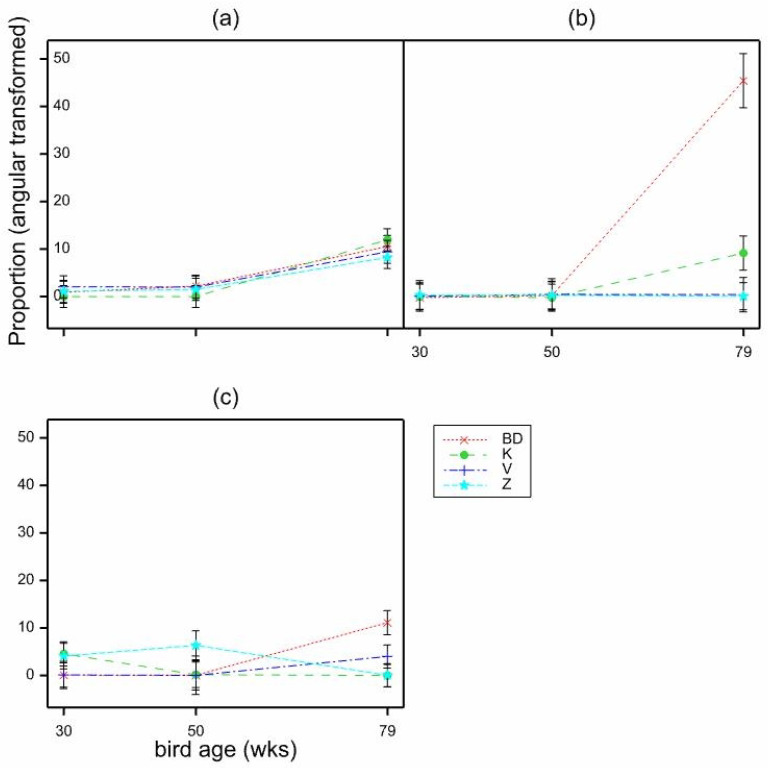
Mean proportions (angular transformed) of cracked eggs per location according to location (laid opposite the (**a**) nest, (**b**) scratch, (**c**) other cage areas), bird age (30, 50 and 79 weeks) and scratch mat type (BD, K, V, Z), estimated from LMM. Mean SE 2.819.

There was no significant effect of location × mat type (*p* = 0.152), but there was a significant 3-way interaction of age × location × mat type (*p* < 0.001, by LMM, F_12,268_ = 3.42, mean SE 2.819), which indicates that the biggest influencer on the proportion of cracked eggs per location at the scratch mat at 79 weeks of age (as seen in Figure 8. The mean proportions (angular transformed) of cracked eggs per location by bird age (weeks) and location in the cage (nest, scratch mat, all other areas) estimated from LMM. Mean SE 1.465) is from Big Dutchman scratch mats (Figure 10).

## 4. Discussion

In this study, only about 2 hens were observed on the mats per observation, and as a result the proportions of birds out of populations of 60 hens/cage on the mats was very low. Although the proportions of hens on the mats was significantly lowest at 79 weeks of age, the differences are small (between 0.02 and 0.03). The lack of an effect of observation on the counts of hens on the mats was surprising, given that more hens were expected on the mats during or shortly after scratch feed application (2nd observation), when the scratch mat is assumed to be at its most attractive, but mat areas (apart from Z) are similar in area to an A4 sheet of paper, which would not be able to accommodate many hens.

High proportions of inactive (stand, sit) behaviours were recorded at the scratch mats, accounting for 0.97 of observations. This may be because hens are genuinely inactive at the mats, or that observer presence disturbed hens from more active behaviours. By contrast, in a study of laying hens in enriched cages with various keel bone fracture severity, stand and sit behaviours accounted for only 23.6–30.0% of behaviours observed, but that was not restricted to the scratch mat area, and hens were in a research environment [22]. Commercial hens may see people less frequently than those in a research facility, so may be less habituated to their presence. Also, in commercial enriched caged systems, aisle widths restrict the distance the observer can be from the area of interest: aisle widths must be at least 90 cm between tiers [1], and to make best use of space, may typically be no more than this. Using remote or automated equipment is one way to avoid bird disturbance [23], however it was not possible to install video equipment to record behaviour at the commercial farm. The larger mat (Zucami) saw a higher proportion of hens on it than any other mat, but the difference in bird proportions is in reality small (i.e., equates to 0.037 of hens in a cage on Z mats versus 0.022–0.023 of hens in a cage on other mats), and in fact, relative to the area, half as many birds were on Z compared to K.

As hens got older, they tended to stand less, and sit more, on the mats. Sitting behaviour was lower, and foraging behaviour was higher (particularly at 50 and 79 weeks of age), at the 2nd observation during or shortly after scratch feed application. The amount of foraging behaviour observed on the mats was generally very low, although it was higher at 50 and 79 weeks of age compared to 30 weeks of age, and dustbathing (which might also be expected to be elicited by litter on the mat) was not observed at all. As with other work [24], it is logical that foraging behaviour is most likely to occur during the presence of litter, however litter is quickly eaten or depleted [25], and thus the positive feedback from foraging also ceases then. Therefore, if foraging behaviour is to be stimulated for longer durations or more frequently, a greater quantity of litter or a higher frequency of provision might need to be provided. The use of layer’s feed as litter should be attractive to hens as a foraging substrate: in a study comparing wood shavings, pelleted lignocellulose, no substrate or layer’s feed, the feed was preferred for foraging [26], whereas a bare mat is not attractive for foraging [26,27]

Most eggs were laid in the nest, which suggests that hens found the nest design suitable for egg laying behaviour over most other areas of the cage [28]. This agrees with previous work where Big Dutchman enriched cages were studied, and those nests were favoured by Hy-Line brown hens for the majority of egg laying [29]. Eggs that were not laid in the nest box were more likely to be found in other areas of the cage rather than opposite the scratch mat. This is in contrast to Hunniford et al. [19], who found that in both small (28 or 40 hens/cage) and large (55 or 80 hens/cage) enriched cages, most eggs laid outside of the nest were laid at the scratch mat. The vast majority of eggs were clean, which is highly desirable in commercial egg production. The proportion of clean eggs declined, and the proportion of dirty eggs increased, particularly at the scratch mat, at 79 weeks of age. Onbaşilar et al. [21] found that a higher percentage of dirty eggs in enriched cages were laid outside of the nest box, but they did not distinguish between eggs laid at the mat and other areas. In cage designs that offered litter either on scratch mats on the wire or in litter boxes above the nest, both egg laying in the litter facility and dirty eggs tended to be higher in cages providing scratch mats [8], however the cage design was also confounded with group size. Here, no mat type was more likely to have dirty eggs than another. It is notable in this study that no mats were made of Astoturf-type material, but instead were hard plastic which may stay clean more easily.

As expected, cracked eggs were greatest when hens were oldest, because eggshell quality declines with hen age as egg size increases and eggshell thickness decreases [30,31]. Of the small proportion of cracked eggs seen, at age 79 weeks they were less likely to be cracked if they were laid in other areas than the nest or scratch mat. This is similar to Onbasilar et al. [21] who found significantly fewer cracked eggs outside rather than in the nest box. This may be because hens on these other areas are closer to the food trough (so eggs have less far to roll) than the nest or scratch mat, which are positioned furthest from the egg belt. Another explanation for this may simply be that since fewer eggs are laid outside the nest box, there are less likely to be collisions between eggs, which can crack one another. There were more likely to be cracked eggs if laid at the BD mat than any other mat type, at 79 weeks of age, but there is no clear reason for this, and it should be kept in mind that cracked eggs overall were low.

## 5. Conclusions

Overall, scratch mat designs studied here did not appear to be major influencers on hen behaviour or egg characteristics. Although the application of litter onto the mats did not increase proportion of hens found there (compared to other observation times), hens at that observation were more likely to be foraging then. Most eggs laid were clean and laid in the nest. It may be that these scratch mat designs are equally adequate (or inadequate) at eliciting behaviours there, or that the study design disturbed behaviour too much to get a true record of what happens at the mats. Further work would benefit from studying behaviour at mats remotely or with human-habituated hens.

## Figures and Tables

**Table 1:** Mat Types (BD = Big Dutchman, K = Kovobel, V = Valli, Z = Zucami) and Their Size (length × width), Total Mat Area and Area per Hen (based on 60 hens per cage, ignoring any cage mortality). Relative Area was Calculated for Later Statistical Analysis.

Mat Type	Length × Width (cm)	Mat Area (cm^2^)	Mat Area/Hen (cm^2^)	Relative Scratch Mat Area
BD	35.0 × 26.5	927.5	15.5	0.214
K	30.5 × 19.0	579.5	9.7	0.134
V	44.8 × 18.0	806.4	13.4	0.186
Z	64.0 × 31.5	2016.0	33.6	0.466

**Table 2:** Ethogram of Behaviours Observed on the Scratch Mats. Any Part of the Bird Touching the Mat Counted as Being ‘on the Mat’.

Behaviour	Description
Stand	At least one foot on the mat, stationary with no other activity
Sit	Sitting on the mat with no other activity
Forage	Pecking and scratching at the mat (feet may be off the mat)
Dustbathe	Dustbathing (or sham dustbathing) on the mat
Preen	Self-grooming on the mat
Walk	Moving across the mat
Other	All other behaviours

**Table 3:** Proportion of Hens, Overall Observations, Performing Each Behaviour on the Scratch Mats.

Stand	Sit	Forage	Dustbathe	Preen	Walk	Other
0.720	0.250	0.021	0.000	0.004	0.005	0.000

**Table 4:** Within Bird Ages (weeks), the Mean Proportion of Observed Eggs Laid by: (**a**) Egg Type (clean, dirty and crack); (**b**) Cage Location (opposite the nest box, scratch mat, all other areas).

(a) Egg Type				(b) Cage Location			
Bird Age	Clean	Dirty	Crack	Bird Age	Nest	Scratch	Other
30	0.991	0.000	0.009	30	0.850	0.052	0.098
50	0.965	0.027	0.008	50	0.878	0.054	0.068
79	0.858	0.094	0.048	79	0.825	0.053	0.138

**Table 5:** Within Cage Location (opposite the nest box, scratch mat, all other areas), the Mean Proportion of Observed Eggs Laid by Egg Type (clean, dirty, crack).

	Cage Location		
Egg Type	Nest	Scratch	Other
clean	0.970	0.895	0.930
dirty	0.011	0.086	0.043
crack	0.019	0.019	0.027

## Data Availability

The data presented in this study are openly available in Zenodo at doi 10.5281/zenodo.4630454, reference number md5:2bbc31c13aaf30f8e56ae2c3b5691b01.
